# Cholbam® and Zellweger spectrum disorders: treatment implementation and management

**DOI:** 10.1186/s13023-021-01940-z

**Published:** 2021-09-14

**Authors:** Janaina Nogueira Anderson, Zineb Ammous, Yasemen Eroglu, Erick Hernandez, James Heubi, Ryan Himes, Sirish Palle

**Affiliations:** 1 Department of Pediatric Gastroenterology, Hepatology, and Nutrition, Medical University of South Carolina, 135 Rutledge Avenue, MSC 561, Charleston, SC 29465 USA; 2 The Community Health Clinic, Topeka, IN USA; 3 Division of Pediatric Gastroenterology, Hepatology, Pancreatology and Nutrition, Stead Family Department of Pediatrics, IU Carver College of Medicine, Iowa City, IA USA; 4 Department of Pediatric Gastroenterology, Hepatology and Nutrition, Pediatric Gastroenterology Associates, Nicklaus Children’s Hospital, Miami, FL USA; 5 Divisions of Pediatric Gastroenterology, Hepatology and Nutrition and Pathology and Laboratory Medicine, Cincinnati Children’s Hospital Medical Center, University of Cincinnati, Cincinnati, OH USA; 6 Division of Pediatric Hepatology, Ochsner Health, New Orleans, LA USA; 7 Department of Pediatric Gastroenterology, Oklahoma University Medicine, Oklahoma City, OK USA

**Keywords:** Zellweger spectrum disorder, Zellweger disease, Peroxisome biogenesis disorder, Hepatic injury, Cholic acid therapy

## Abstract

**Background:**

Zellweger spectrum disorders (ZSDs) are a rare, heterogenous group of autosomal recessively inherited disorders characterized by reduced peroxisomes numbers, impaired peroxisomal formation, and/or defective peroxisomal functioning. In the absence of functional peroxisomes, bile acid synthesis is disrupted, and multisystem disease ensues with abnormalities in the brain, liver, kidneys, muscle, eyes, ears, and nervous system.

**Main body:**

Liver disease may play an important role in morbidity and mortality, with hepatic fibrosis that can develop as early as the postnatal period and often progressing to cirrhosis within the first year of life. Because hepatic dysfunction can have numerous secondary effects on other organ systems, thereby impacting the overall disease severity, the treatment of liver disease in patients with ZSD is an important focus of disease management. Cholbam® (cholic acid), approved by the U.S. Food and Drug Administration in March 2015, is currently the only therapy approved as adjunctive treatment for patients with ZSDs and single enzyme bile acid synthesis disorders. This review will focus on the use of CA therapy in the treatment of liver disease associated with ZSDs, including recommendations for initiating and maintaining CA therapy and the limitations of available clinical data supporting its use in this patient population.

**Conclusions:**

Cholbam is a safe and well-tolerated treatment for patients with ZSDs that has been shown to improve liver chemistries and reduce toxic bile acid intermediates in the majority of patients with ZSD. Due to the systemic impacts of hepatic damage, Cholbam should be initiated in patients without signs of advanced liver disease.

## Background

When the liver is confronted with chronic injury, the result is often hepatic fibrosis, which involves the excess deposition of extracellular matrix and production of fibrous connective tissue as a reparative process to contain the site of injury [1]. Formation of fibrous scar tissue distorts the hepatic architecture and, in the presence of continued insult, subsequently leads to cirrhosis. Chronic injury leading to fibrosis may occur in response to a number of insults, among which are genetic disorders [2]. The Zellweger spectrum disorders (ZSDs) represent one such genetic disorder that may result in hepatic fibrosis early in the postnatal period [3–5].

ZSDs are a rare, heterogeneous group of autosomal recessively inherited disorders characterized by defective peroxisomes [3, 4]. These disorders are caused by pathogenic sequence variants in one of 13 different *PEX* genes encoding peroxins, which are involved in peroxisome formation and/or peroxisomal protein import [4]. Peroxisomes, which are subcellular organelles present in all eukaryotic cells, catalyze a number of essential metabolic functions, including the α- and β-oxidation of very long-chain, branched-chain, and dicarboxylic fatty acids, the biosynthesis of plasmalogens and bile acids, and the detoxification of glyoxylate and reactive oxygen species (ROS) [4–6]. The ZSDs encompass a spectrum of disease symptoms and severity, with phenotypes ranging from mild (infantile Refsum disease, IRD) and intermediate (neonatal adrenoleukodystrophy, NALD) to severe (Zellweger syndrome, ZS) [7]. Common symptoms include craniofacial dysmorphism, cognitive defects, visual impairment and sensorineural hearing loss, liver dysfunction, adrenal insufficiency, and renal stones [4, 8]. Patient survival varies with disease severity: patients with mild ZSD survive into adulthood, patients with moderate ZSD tend to survive into their teens, and patients with severe ZSD typically do not survive beyond their first year [9, 10].

**Fig. 1:**
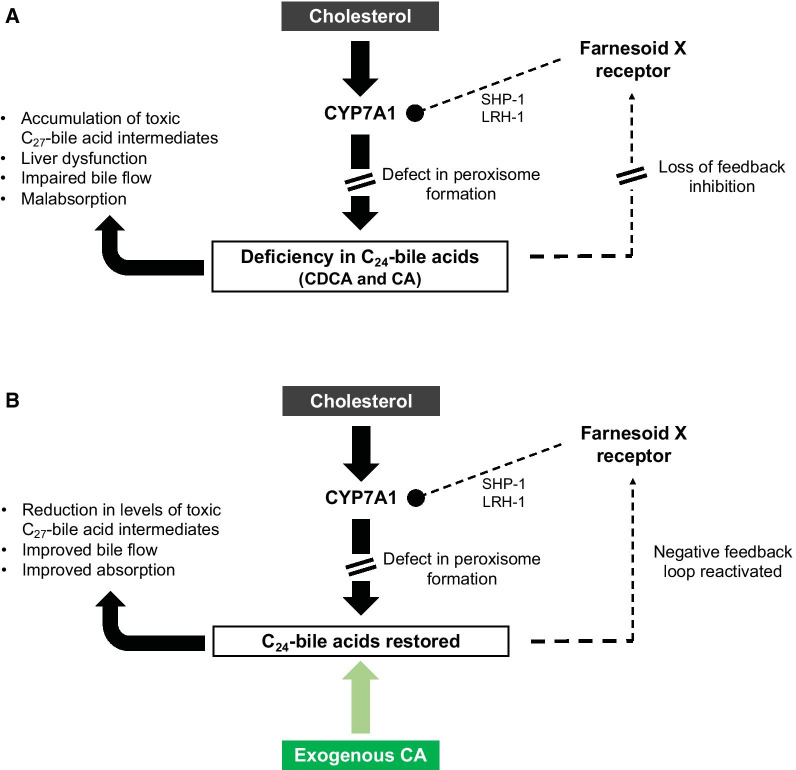
Disrupted bile acid synthesis in patients with ZSD and CA mechanism of action. CA, cholic acid; CDCA, chenodeoxycholic acid; CYP7A1, cytochrome P450 7A; LRH-1, liver receptor homolog 1; SHP-1, small heterodimeric partner 1. **A** Disrupted bile acid synthesis in patients with ZSD. In the absence of functional peroxisomes, there is a deficiency of primary C_24_-bile acids, which subsequently results in the disruption of the negative feedback loop (dotted lines) that maintains bile acid homeostasis under normal physiological conditions. Increased transcription of CYP7A1 causes a build-up of C_27_-bile acid intermediates, which have been shown to be cytotoxic. Additionally, deficiency in primary bile acids causes impaired bile flow and cholestasis, as well as reduced absorption of dietary fats and fat-soluble vitamins. **B** CA mechanism of action in patients with ZSD. Introduction of exogenous CA restores levels of the primary bile acids, improves bile flow and absorption, and reactivates the negative feedback loop, which, via repression of CYP7A1, reduces levels of the C_27_-bile acid intermediates. Adapted from Gonzales et al. [13], with permission from Elsevier

As previously indicated, peroxisomes play an important role in the biosynthesis of bile acids from cholesterol in the liver. The final steps of bile acid synthesis occur in the peroxisome, where the side chains of the C_27_-bile acid intermediates dihydroxycholestanoic acid (DHCA) and trihydroxycholestanoic acid (THCA) are shortened by β-oxidation, resulting in the formation of the mature C_24_-bile acids chenodeoxycholic acid (CDCA) and cholic acid (CA) [11]. In the absence of functional peroxisomes, there is a reduction of C_24_-bile acids, as well as a build-up of the C_27_-bile acid intermediates, which have been shown to be cytotoxic (Fig. 1A) [11, 12]. Ferdinandusse et al. [12] demonstrated that incubation with increasing concentrations of C_27_-bile acid intermediates resulted in a dose-dependent decrease in cell viability, decrease in ATP synthesis, and stimulation of ROS generation in the rat hepatoma cell line McA-RH7777. Additionally, because the CoA esters of DHCA and THCA are poor substrates for the glycine/taurine-conjugating enzyme bile acid: amino acid *N*-acyltransferase (BAAT), these bile acid intermediates are present mainly in unconjugated forms, which are poorly excreted into the canalicular space and result in increased hepatotoxicity [5]. Taken together, these findings suggest that increased production of the toxic C_27_-bile acid intermediates DHCA and THCA are an important contributing factor to the liver disease associated with ZSDs.

Treatment of liver disease in ZSD is a complex challenge involving the totality of the patient’s condition, extent of liver involvement, and potential for therapeutic benefit. This challenge is further complicated by a lack of robust natural history data supporting expectations of treatment outcome and by conflicting efficacy and safety data. Cholbam® (cholic acid) is the only U.S. Food and Drug Administration (FDA)-approved therapy for the treatment of ZSDs in both pediatric and adult patients who exhibit liver disease, steatorrhea, or complications from reduced absorption of fat-soluble vitamins [14]. In this review, recommendations for appropriate administration of CA will be provided, including the best practices for initiating and maintaining CA therapy.

## CA mechanism of action

Bile acids play several important roles in the liver, including transport of nutrients and drugs to the liver for metabolism, stimulation of bile flow and enterohepatic circulation, and absorption of lipid-soluble vitamins, dietary fats, and sterols in the intestine. Additionally, bile acids regulate hepatic lipid, glucose, and energy metabolism via activation of the nuclear bile acid receptor farnesoid X receptor (FXR) and the membrane G protein-coupled receptor TGR5 [15]. Given their essential role in many physiological processes, as well as the fact that several bile acid intermediates are cytotoxic, bile acid biosynthesis needs to be tightly controlled. Under normal physiological conditions, there exists a negative feedback loop that keeps levels of bile acid intermediates in the liver low to prevent their deleterious effects on intracellular processes [5]. The C_24_-bile acids CDCA and CA bind to FXR, which activates the transcription of short heterodimeric partner 1 (SHP-1). SHP-1 then inhibits transcription of liver receptor homolog 1 (LRH-1), which normally transactivates cytochrome P450 7A (CYP7A1), the rate-limiting enzyme for bile acid synthesis [5, 16]. In the absence of functional peroxisomes, as in ZSD, the defective production of mature C_24_-bile acids disrupts the negative feedback loop, causing accumulation of toxic C_27_-bile acid intermediates (Fig. 1A) [17].

Based on this pathway, CA therapy has been the subject of a number of studies in ZSD. It is hypothesized that introduction of exogenous CA will:

1. Reduce levels of the toxic C_27_-bile acid intermediates via restoration of the negative feedback loop and subsequent downregulation of CYP7A1 (Fig. 1B), resulting in reduction in the level of toxic C_27_-bile acid intermediates, which will reduce injury to the liver, limiting hepatic disease progression, as well as extrahepatic injury, which may lead to central nervous system (CNS) dysfunction [17–19]. It has been theorized that DHCA and THCA cross the blood–brain barrier to mediate effects on the CNS; however, the process by which this occurs is not well understood [4].
2. Restore C_24_-bile acid levels, thereby improving bile flow, which will facilitate biliary excretion of bile acids and improve cholestasis (Fig. 1B) [17–19].
3. Increase intraluminal bile acid concentration, which will facilitate the absorption of dietary fats and fat-soluble vitamins and potentially improve growth [17–19].

Oral bile acid therapy has been tested in animal models of ZSD, most notably the *PEX2* knockout mouse [11]. *PEX2*^*−/−*^ mice have a marked deficiency of peroxisomal assembly characterized by reduced C_24_-bile acids, accumulated C_27_-bile acid intermediates, and low total bile acid levels in both liver and bile. These mice have severe intrahepatic cholestasis that abates in the early postnatal period and progresses to steatohepatitis by postnatal day 36. Treatment of *PEX2*^*−/−*^ mice with a mixture of CA and ursodeoxycholic acid (UDCA) starting on postnatal day 1 improved postnatal survival, increased body fat deposition with significant resolution of steatorrhea, alleviated intrahepatic cholestasis, reduced C_27_-bile acid intermediate production, and prevented older mutants from developing severe steatohepatitis. Notably, however, other aspects of the liver worsened in treated mice, including steatosis and mitochondrial and cellular damage, suggesting that bile acid therapy has limited effectiveness in preventing hepatic disease [11]. The use of the combination of UDCA and CA in these analyses may have blunted the potential value of CA since UDCA may have competitively inhibited the absorption of CA and thereby reduced bile acid pool enrichment with CA and inhibited its role in feedback inhibition of Cyp7a and reduction in potentially toxic metabolites, THCA and DHCA.

## Clinical studies demonstrating efficacy of CA therapy in patients with ZSD

At present, clinical data supporting the efficacy of CA therapy in patients with ZSD are limited (Table 1). The initial observation of its utility was described in a case report of a 2-month-old boy who presented with the typical signs of ZS, including marked craniofacial dysmorphism, severe hypotonia, hearing and retinal abnormalities, seizures, and hepatomegaly with signs of liver dysfunction [18]. Liver biopsy demonstrated the absence of well-formed peroxisomes. At 28 weeks of age, the patient was started on 100 mg/day each of CA and CDCA. A significant improvement in biochemical indices of liver function occurred along with a histological decrease in the extent of bile duct proliferation and inflammation and a normalization of serum bilirubin and liver enzymes, including alanine aminotransferase (ALT) and γ-glutamyl transpeptidase. Importantly, plasma and urinary levels of C_27_-bile acid intermediates decreased with treatment. Steatorrhea improved and was accompanied by a marked increase in growth. Neurological symptoms improved, including reduction in seizures and improved spontaneous motility. Despite the observed improvements, 8 months after initiating treatment, the patient died at one year of age with respiratory failure [18].

**Table 1:** Summary of key clinical studies of CA therapy in patients with ZSD

Study	Design	Patients	CA dosage	Study duration	Major findings	Conclusions	Limitations
Berendse et al*.* [17]	Open-label, pretest-postest study	19 patients with ZSD (mean age 14 years)	15 mg/kg/day (with dose adjustments, as needed)	36 weeks	Signficant increases in plasma CA levels Signficant decreases in plasma levels of C_27_-bile acid intermediates No changes in: AST, ALT, or conjugated bilirubin Fibroscan® liver stiffness values Concentrations of fat-soluble vitamins Weight Subset of patients with advanced liver disease at baseline (n = 4) showed evidence of increased liver damage with CA therapy	CA therapy can be used in the majority of patients with ZSD, leading to at least partial supression of bile acid synthesis Caution is needed in patients with advanced liver disease because of possible hepatotoxic effects	Small size of the cohort Short duration of treatment Mild disease severity at start of treatment
Klouwer et al*.* [20]	Extension of Berendse et al*.* 2016 pretest–posttest study	22 patients with ZSD (mean age 13 years)	15 mg/kg/day (with dose adjustments, as needed)	Additional 12 months (total duration 21 months)	Significant decreases in plasma C_27_-bile acid intermediates No changes in: AST, ALT, or conjugated bilirubin Liver elasticity Concentrations of fat-soluble vitamins Weight Subset of patients with advanced liver disease at baseline (n = 6) had progressive increases in conjugated bilirubin levels with CA therapy	Although CA therapy resulted in lower C_27_-bile acid intermediate levels in plasma and urine, no clincial benefit was observed CA therapy can be harmful to ZSD patients with cirrhosis	Small size of the cohort Short duration of treatment Mild disease severity at start of treatment
Heubi et al*.* [19]	Phase 3, open-label, single-arm, nonrandomized, noncomparative study	70 patients SED, n = 50 ZSD, n = 20 (mean age 3 years)	10–15 mg/kg/day	18 years	Significant improvements in FAB-MS scores Significant reductions in AST, ALT, and serum direct bilirubin levels Significantly improved weight profiles Improvement in nearly all histological parameters TEAEs generally mild to moderate	CA therapy is an effacious, safe, and well-tolerated treatment for patients with ZSD	Lack of a placebo control group Collection of laboratory data and urine FAB-MS was not defined at specific and rigid times during the study period
Heubi and Setchell [21]	Phase 3, open-label, single-arm extension study of Heubi et al*.* [19]	53 patients (31 previously treated, 22 treatment naïve) SED, n = 41 ZSD, n = 12 (mean age 9 years)	10–15 mg/kg/day	Additional 6 years	Significant improvements in urinary bile acids, height, and weight AST and ALT levels decreased in treatment-naïve patients and remained stable in previously treated patients TEAEs predominantly mild to moderate	Findings reinforce the short-term (in treatment-naïve patients) and long-term (in previously treated patients) efficacy and safety of CA therapy in patients with ZSD	Lack of systematic follow-up Small number of treatment-naïve patients with ZSD Use of urine vs serum for assessing DHCA and THCA may have compromised ability to detect changes with treatment in patients with ZSD

*ALT* alanine aminotransferase, *AST* aspartate aminotransferase, *CA* cholic acid, *DHCA* 3α,7α- dihydroxycholestanoic acid, *FAB-MS* fast atom bombardment ionization mass spectrometry, *SED* single enzyme defect, *TEAE* treatment-emergent adverse event, *THCA* 3α,7α,12α-trihydroxycholestanoic acid, *ZSD* Zellweger spectrum disorder

The case study results reported by Setchell et al*.* formed the basis for a larger cohort study of CA therapy in patients with ZSD. In this open-label, pretest–posttest study, 19 patients (aged 2–35 years) with genetically confirmed ZSD were treated with 15 mg/kg/day of CA for 36 weeks [17]. The dose was increased to 20 mg/kg/day in five patients at different time points because of persistently elevated plasma C_27_-bile acid intermediates, and it was decreased to 10 mg/kg/day in four patients because of diarrhea (2 patients), an increase in plasma transaminases (1 patient), and an increase in conjugated bilirubin (1 patient). After 4, 12, and 36 weeks of CA supplementation, there was a significant increase in plasma CA levels, as well as a significant decrease in the levels of the C_27_-bile acid intermediates DHCA and THCA. Furthermore, plasma levels of fibroblast growth factor 19 (FGF19), a negative regulator of CYP7A1 expression, were increased, whereas plasma levels of C4, a marker for CYP7A1 enzyme activity, were decreased, both of which indicated suppression of bile acid synthesis. No changes from baseline in median aspartate aminotransferase (AST), ALT, and conjugated bilirubin levels; Fibroscan® liver stiffness values; fat-soluble vitamin (A, E, and D) concentrations; or weight were observed with CA supplementation. As a post hoc analysis, the patients were divided into two subgroups based on the degree of liver stiffness prior to therapy, as assessed by Fibroscan® analysis: group 1, Fibroscan® < 15.5 kPa (n = 15) and group 2, Fibroscan® ≥ 15.5 kPa (n = 4). Interestingly, the decrease in DHCA and THCA following CA supplementation was observed in only group 1, not group 2. Similarly, the changes in FGF19 and C4 levels were also observed in only group 1. The patients in group 2 also had markedly increased plasma CA levels upon CA supplementation, as well as an increase (albeit not statistically significant) in plasma transaminases and conjugated bilirubin, suggesting advanced liver damage [17].

**Fig. 2:**
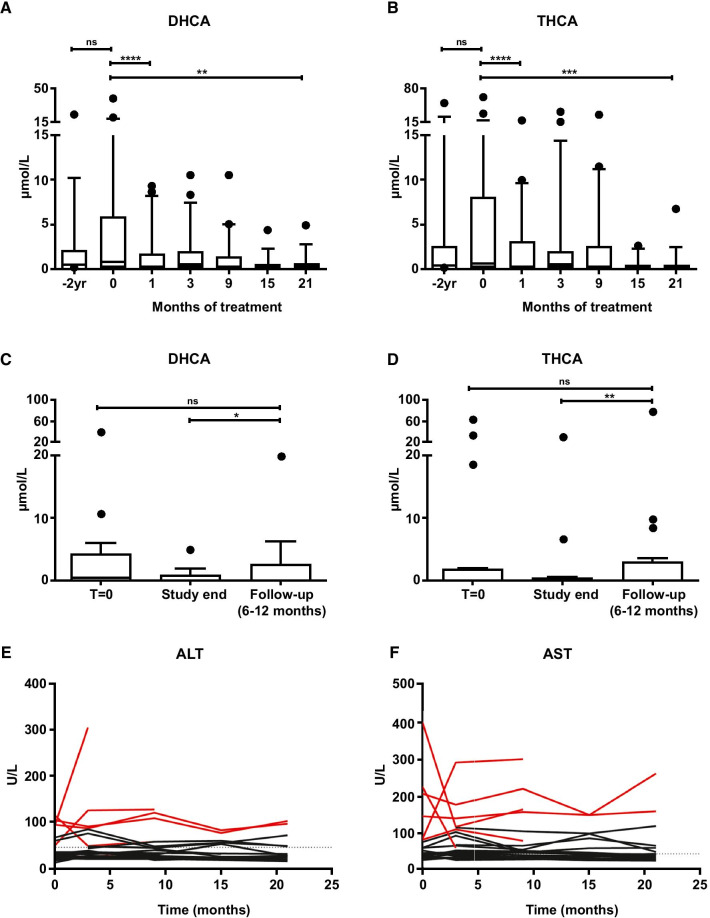
Klouwer et al. [20]: impact of cholic acid therapy on bile acid levels, and liver chemistry. **A**, **B** Tukey box plots showing the effect of oral cholic acid (CA) on plasma 3α,7α- dihydroxycholestanoic acid (DHCA) and 3α,7α,12α-trihydroxycholestanoic acid (THCA) after 1, 3, 9, 15 and 21 months of treatment. The control reference range for THCA is < 0.05–0.1 μmol/L and levels of DHCA are undetectable (< 0.05 μmol/L) in control individuals. **C**, **D** Tukey box plots showing the levels of plasma DHCA and THCA at baseline, study end (after 9 or 21 months of CA treatment) and at follow-up (6–12 months after discontinuation of CA). Only the levels of patients for which follow-up values were available are shown (n = 16). **E**, **F** Graphs showing the individual courses of alanine transaminase (ALT) and aspartate transaminase (AST) levels during oral CA treatment (n = 22). The patients with liver cirrhosis are depicted in red. The upper control reference ranges of ALT (40 U/L) and AST (45 U/L) are indicated by the dotted lines. *P<0.05, **P<0.01, ***P<0.005, ****P<0.001, ns, not significant. Reprinted from Klouwer et al. [20]; with permission from John Wiley and Sons. This is an open access article distributed under the terms of the Creative Commons CC BY license, which permits unrestricted use, distribution, and reproduction in any medium, provided the original work is properly cited

An extension of this study was conducted for an additional 12 months in 22 patients with ZSD [20]. The 19 patients continuing from the initial study were maintained on the same dose that they were on at the conclusion of the 9-month treatment phase. Patients who were not included in the initial 9-month treatment phase were started with a dose of 15 mg/kg/day. Again, the dose was increased to 20 mg/kg/day when patients demonstrated persistently elevated plasma C_27_-bile acid intermediates, and it was decreased to 10 mg/kg/day when adverse events, such as diarrhea, vomiting, or worsening liver tests, occurred. After 3, 9, 15, and 21 months of CA supplementation, median plasma levels of DHCA and THCA significantly decreased (Fig. 2A–D), median FGF19 levels significantly increased, and median C4 levels significantly decreased compared with median baseline levels, indicating suppressed bile acid synthesis. Similar to the findings of Berendse et al*.* [17], no changes from baseline in median AST, ALT, and conjugated bilirubin levels; liver elasticity values; fat-soluble vitamin (A, E, and D) concentrations; or weight were observed with CA supplementation (Fig. 2E, F) [20]. Patients were divided into two subgroups, those with (n = 6) and those without (n = 16) severe liver fibrosis or cirrhosis, based on ultrasound and/or elastography values ≥ 15.5 kPa, and significant differences in baseline levels of FGF21, an endocrine factor that becomes elevated under conditions of stress in the liver, were found. Compared with the 16 patients without severe liver fibrosis/cirrhosis, the 6 patients with severe liver fibrosis/cirrhosis had markedly elevated baseline FGF21 levels. During CA supplementation, these same patients had elevated baseline levels of conjugated bilirubin, as well as a progressive increase in conjugated bilirubin levels. Based on the results of the initial 9-month treatment period and the 12-month extension study, the authors concluded that CA therapy may be effective in a subset of patients with ZSD, leading to a partial suppression of bile acid synthesis in the majority of patients. However, in patients with advanced liver disease, CA therapy may have been hepatotoxic; thus, the authors cautioned against its use in patients with a more severe liver phenotype [17, 20]. Based upon experience in patients with ZSD, it is clear that patients with advanced disease may not benefit from CA therapy and progress despite its use. However, anecdotal evidence from patients with single-enzyme defects suggest that CA, if started rapidly after diagnosis, is effective even in patients with severe liver disease and fibrosis, distinguishing patients with advanced damage due to single-enzyme deficiency from counterparts with ZSD [19].

**Fig. 3:**
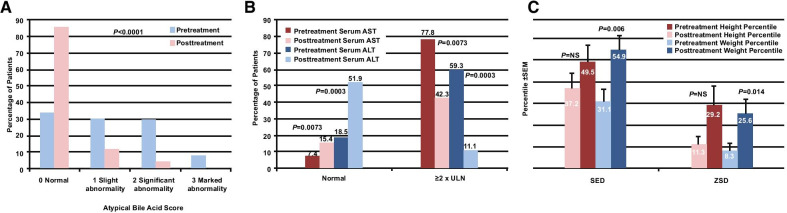
Heubi et al. [19]: impact of cholic acid therapy on bile acid levels, liver chemistry, and height and weight. ALT, alanine transaminase; AST aspartate transaminase; NS, not significant; SED, single enzyme defect; SEM, standard erroor of the mean; ULN, upper limit of normal. **A** Impact of cholic acid treatment on urinary bile acid excretion patients with Zellweger spectrum disorder (ZSD) (n = 27)—worst-to-best analysis, modified intent-to-treat (mITT) population. **B** Impact of cholic acid treatment on liver chemistries in patients with ZSD (n = 27)—worst-to-best analysis, mITT population. **C** Mean height and weight percentiles from pretreatment to post-treatment in the mITT population (N = 70), worst-to-best analysis. Numbers in bars represent absolute percentiles for each group. Reprinted from Heubi et al. [19]. This is an open access article distributed under the terms of the Creative Commons CCBY-NC-ND license, where it is permissible to download and share the work provided it is properly cited. The work cannot be changed in any way or used commercially without permission from the journal

Results from the largest clinical study to date investigating the use of CA in patients with a bile acid synthesis disorder led to the FDA approval of Cholbam (CA) in March 2015 [14, 19]. In this phase 3, open-label, single-arm, nonrandomized, noncomparative study conducted over 18 years, the efficacy and safety of oral CA were evaluated in 70 patients with either a single enzyme defect (SED; n = 50) or ZSD (n = 20) [19]. ZS (median age, 6 years) and NALD (median age, 2 years) were the most common ZSDs. Patients were administered 10–15 mg/kg/day of CA once daily or in divided doses twice daily as capsules emptied into food or as a liquid formulation (15 mg/mL) for patients who could not swallow capsules. The mean duration of treatment for patients in the safety set was 145 weeks (range 0–545 weeks). Treatment with CA significantly improved urine bile acid fast atom bombardment ionization mass spectrometry (FAB-MS) scores in patients with SED and ZSD, indicating improved urinary bile acid excretion. Among patients with ZSD, the percentage with normal FAB-MS scores increased from 33.3% to 85.2% (*P* < 0.0001) with CA treatment (Fig. 3A). CA treatment significantly improved liver chemistries, with a marked increase in the number of SED and ZSD patients with serum ALT and AST values below the upper limit of normal (ULN) and a marked decrease in the number with values ≥ 2 times the ULN (*P* < 0.0001). The majority of patients with ZSD had elevated AST (77.8%) and ALT (59.3%) levels at baseline. Posttreatment, 42.3% had elevated AST levels (*P* = 0.007), and 11.1% had elevated ALT levels (*P* = 0.0003), suggesting a reduction in biochemical markers of liver disease (Fig. 3B). Serum direct bilirubin also significantly decreased from 3.5 to 0.6 mg/dL (*P* < 0.001). CA treatment significantly improved weight profiles for both patients with SED (*P* = 0.006) and those with ZSD, with the weight percentiles of patients with ZSD increasing from 8.3% pretreatment to 25.6% posttreatment (*P* = 0.014; Fig. 3C). Furthermore, with the exception of bridging fibrosis, all histological parameters improved with CA treatment, including cholestasis, giant cells, and necrosis. Treatment-emergent adverse events (TEAEs) were predominantly mild to moderate. Of the 28 serious adverse events (SAEs) reported, the most frequent were disease progression, diarrhea (3%), urinary tract infection (3%), and dehydration (3%). None of the SAEs were considered treatment related. The authors concluded that orally administered CA at a dosage of 15 mg/kg/day is an efficacious, safe, and well-tolerated treatment for patients with an SED or a ZSD [19].

**Fig. 4:**
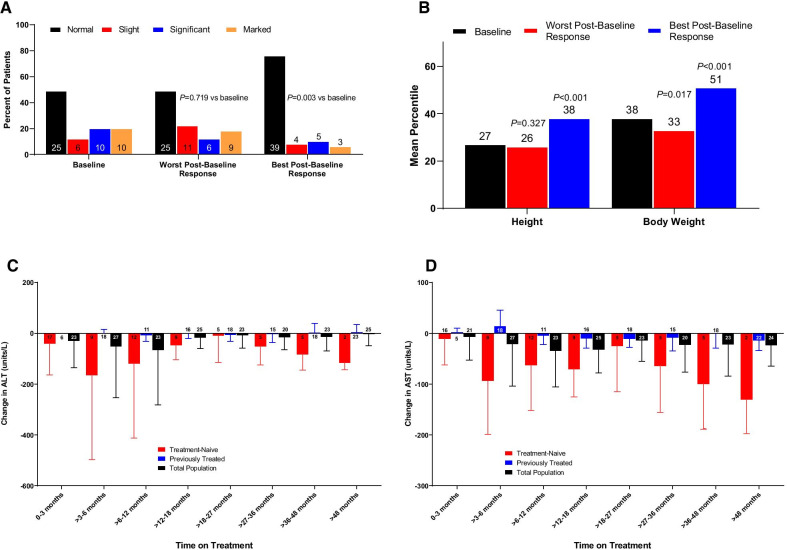
Heubi et al. [21]: impact of cholic acid therapy on bile acid levels, liver chemistry, and height and weight. ALT, alanine transaminase, AST, aspartate transaminase. **A** Urinary bile acids at baseline, worst postbaseline response, and best postbaseline response (total population). **B** Height and body weight: baseline, worst postbaseline response, and best postbaseline response (total population). Change from baseline over time in **C** serum alanine aminotransferase and **D** serum aspartate aminotransferase. In panels **A**, **C**, and **D**, numbers within the bars indicate n values. Reprinted from Heubi and Setchell [21]. This is an open access article distributed under the terms of the Creative Commons CCBY-NC-ND license, where it is permissible to download and share the work provided it is properly cited. The work cannot be changed in any way or used commercially without permission from the journal

A continuation of this study was undertaken for an additional 6 years to further evaluate the efficacy and safety of CA therapy in patients with a bile acid synthesis disorder [21]. The phase 3, open-label, single-arm extension study included a total of 53 patients, of which 31 (58%) were on CA from the previous study and 22 (42%) were newly diagnosed and treatment naïve. Twelve patients (23%) had a ZSD, and the remaining 41 (77%) had SEDs. Patients were treated orally with 10–15 mg/kg/day of CA. The median duration of treatment for the total population was 1517 days (range 1–2404 days). Using baseline-to-best post-baseline analyses, statistically significant improvements were observed in urinary bile acids (*P* = 0.003), height (*P* < 0.001), and body weight (*P* < 0.001) with CA treatment (Fig. 4A, B). Serum AST and ALT levels tended to decrease from baseline in treatment-naïve patients and remained stable in previously treated patients (Fig. 4C, D). TEAEs were predominantly mild or moderate in intensity, and the majority were considered unrelated to the study treatment. The most common TEAE was upper respiratory tract infection (17%), followed by abdominal pain, diarrhea, disease progression, and reduced serum 25-hydroxyvitamin D (11% each). The overall baseline-to-best results reinforce the findings of the previous study demonstrating efficacy of CA in treatment-naïve patients, as well as indicating an increase in efficacy with long-term CA supplementation in previously treated patients with ZSD [21].

In support of the findings of Heubi et al*.*, recent case reports have demonstrated the long-term efficacy of CA treatment in patients with ZSD, including those presenting with evidence of advanced liver disease at diagnosis. The case studies included 4 pediatric patients who were all diagnosed between 3 and 15 months of age with mild (2 patients) or severe (2 patients) ZSD [9, 22]. Three of these patients, including one who presented with bridging fibrosis at diagnosis, have been maintained on CA therapy for ≥ 15 years; therapy is ongoing, and the patients have attained a good quality of life, including attendance at school with classroom accommodations [22]. The fourth patient, who presented with moderate fibrosis at diagnosis, was successfully maintained on CA therapy for 18 years and 6 months, but eventually died at 19 years of age from hepatocellular carcinoma [9]. In all four patients, ongoing CA treatment was associated with improved or stabilized liver function, as demonstrated by serum biochemistries and liver histology [9, 22]. While these case studies serve to demonstrate the potential long-term benefits of CA therapy, they also reinforce the importance of early intervention with CA before liver disease progresses to an advanced stage.

## Treatment initiation and maintenance

The clinical studies undertaken by Heubi et al*.* [19] and Klouwer et al*.* [20] represent the two largest, systematic clinical trials investigating the use of CA therapy in patients with bile acid synthesis disorders to date. While the findings and conclusions drawn seemingly conflict, the differences may be explained, at least in part, by the differences in the study populations from the 2 cohorts and the study designs. These differences additionally serve to underscore a few important considerations in the treatment of ZSD patients with CA therapy.

The study populations in Heubi et al*.* [19] and Klouwer et al*.* [20] differ in hepatic involvement, disease severity, and age at initiation of CA therapy. The majority of patients with ZSD in the Heubi study had baseline ALT and AST levels ≥ 100 U/L (59.3% and 77.8%, respectively) vs few patients in the Klouwer study (4.5% and 15%, respectively) [19, 20]. Given that the majority of patients with ZSD in the Klouwer study already had normal AST and ALT levels at baseline, it is unlikely that significant improvements in liver chemistries with CA therapy would be demonstrated in this study population; however, liver chemistries did not worsen over the course of treatment (Fig. 2E, F). This hypothesis is supported by the findings of Heubi et al*.* [21], in which serum AST and ALT levels improved in treatment-naïve patients but remained stable in previously treated patients (Fig. 4C, D). The populations also differed in disease severity at initiation of CA therapy. In the Klouwer study, 6 of the 22 patients with ZSD had severe liver fibrosis or cirrhosis at baseline. This subset of patients also had elevated conjugated bilirubin levels in plasma at baseline, with a progressive increase in these levels during the treatment course. According to the prescribing information for Cholbam, worsening liver function is indicative of a failure of treatment, and cessation of therapy is warranted [23]. It is possible that, in these patients, the disease is so advanced that CA therapy is not effective. Patients in the Klouwer study that had normal liver function and no evidence of cirrhosis at baseline generally remained stable for the duration of the study. It is unclear whether this was due to CA therapy or due to the natural history of their liver disease. Lastly, the mean age of patients at initiation of CA therapy in the Heubi study was 3 years vs a mean age of 13 years in the Klouwer study, suggesting a need for early intervention in patients with ZSD. Liver dysfunction in patients with ZSD has considerable impact on other organ systems; therefore, intervention before liver dysfunction becomes profound has the greatest potential to reduce disease severity.

The differences in findings between Heubi et al*.* [19] and Klouwer et al*.* [20] may also be explained by the duration of the studies. Klouwer treated patients for slightly less than 2 years, whereas Heubi reported the long-term outcomes of patients treated over a span of 18 years [19, 20]. A treatment period of 21 months may be too short to conclude whether CA has an effect on clinical progression. The Klouwer study failed to demonstrate an important feature of CA therapy, which is improvement in growth. In contrast, Heubi et al*.* [19] reported a significant improvement in the weight percentiles of ZSD patients treated with CA (Fig. 3C).

The differences in the study populations and design and in the subsequent therapeutic responses between Heubi et al*.* [19] and Klouwer et al*.* [20] suggest an approach to CA therapy that is dependent on the extent of liver damage when treatment is under consideration:

- Cirrhosis with or without decompensation: If CA is used, frequent monitoring for worsening of liver disease is warranted, and treatment should be stopped with any deterioration in condition. Caution should be exercised in patients with cirrhosis, as in these patients CA therapy may not be efficacious; severe cases of hepatotoxicity have been observed in some patients with cirrhosis. Therefore, exacerbation of liver impairment by CA cannot be ruled out.
- No cirrhosis, but evidence of hepatic injury or risk of liver injury (elevations in liver enzymes or atypical bile acids): CA therapy has been associated with improved liver chemistries, reduced atypical bile acids, and improved height and weight.
- No evidence of hepatic involvement (normal liver chemistry, no atypical bile acid levels present): CA is approved for the treatment of ZSD in patients with manifestations of liver disease, steatorrhea, or complications from decreased fat-soluble vitamin absorption. The efficacy of CA therapy in mitigating or reversing liver disease in patients with ZSD is not proven, and its use should be considered on a case-by-case basis, possibly from a nutritional perspective.

Given that ZSD is a rare disease with no cure, the goal of treatment is to manage symptoms and improve the quality of life of patients and their caregivers. ZSDs are clinically heterogeneous, with high morbidity in most patients and mortality in some, so it is important to help patients and caregivers understand the likely long-term outcomes based on the severity of disease at diagnosis [4]. Furthermore, patients and caregivers should understand how CA therapy may affect prognosis, particularly as a supportive therapy that may help preserve hepatic function. The recommended dosage of CA is 10–15 mg/kg [23]; however, many physicians initiate therapy with a low dose, titrating up to a higher dosage as needed. Certain parameters indicative of effective treatment, such as increases in height and weight, may require longer periods of treatment before they become apparent; therefore, continued treatment may be required to observe effects when those improvements are not immediately apparent.

## Limitations and knowledge gaps

The natural history of liver disease in ZSD is poorly understood. Infants may present with advanced liver disease that progresses to liver failure in the first months of life, yet there are patients who have ongoing disease for years with stabilized liver function and a relatively good quality of life. Thus, a significant limitation in the interpretation of clinical data is the broad spectrum of clinical phenotypes and associated biochemical variations within these cohorts [4]. To this end, a longitudinal natural history study of patients with peroxisome biogenesis disorders (PBDs) is currently underway, and results are expected in early 2022 (clinicaltrails.gov: NCT01668186). Furthermore, new biochemical markers that correlate with disease progression are necessary [4]. Available natural history data and access to improved biochemical markers of disease would be helpful assets in determining the implementation of CA therapy, as well as serving as a baseline of comparison for CA therapy effects over time.

Data from structured, large-scale, long-term clinical studies are scarce. Future studies, particularly those in patients with early initiation of CA therapy, are needed to elucidate the potential long-term hepatic and extrahepatic effects of treatment [9]. These studies would help establish the durability of CA therapy in the maintenance of liver function in patients with ZSD and could determine whether there is a threshold for when treatment may become less able to preserve function. Moreover, future studies should aim to identify whether there is a subgroup of patients with ZSD that would most benefit from CA therapy with regards to age and varying histological and biochemical parameters at diagnosis.

## Summary/conclusions

Cholbam, an oral CA therapy approved by the US FDA in March 2015, is an efficacious, safe, and well-tolerated treatment for bile acid synthesis disorder due to single enzyme defects, and for adjunctive treatment of peroxisomal disorders, including ZSD, in patients who exhibit manifestations of liver disease, steatorrhea, or complications from decreased fat-soluble vitamin absorption [23]. For the majority of patients with ZSD, CA therapy has been shown to improve abnormal liver chemistries and histology, reduce levels of the toxic C_27_-bile acid intermediates DHCA and THCA in plasma and urine, stabilize or reduce hepatic inflammation and fibrosis, and improve growth. The approach to CA therapy in patients with ZSDs should be dependent on the extent of liver damage when treatment is under consideration, with patients who show signs of liver damage without progression to cirrhosis being the most likely to benefit from treatment. In ZSD patients with advanced liver disease, caution should be used in administering CA therapy because, in this subset, CA therapy may not be efficacious; in these patients, frequent monitoring of liver function is warranted to help identify a failure of treatment. For patients with ZSD, the use of CA should be as a supportive therapy to help manage symptoms and improve quality of life.

## Data Availability

Any information relating to the review can be provided upon request.

## Abbreviations

ALT: Alanine aminotransferase
AST: Aspartate aminotransferase
BAAT: Bile acid: amino acid *N*-acyltransferase
CA: Cholic acid
CDCA: Chenodeoxycholic acid
DHCA: Dihydroxycholestanoic acid
FAB-MS: Fast atom bombardment ionization mass spectrometry
FDA: Food and Drug Administration
FGF: Fibroblast growth factor
FXR: Farnesoid X receptor
IRD: Infantile Refsum disease
LRH-1: Liver receptor homolog 1
NALD: Neonatal adrenoleukodystrophy
PBD: Peroxisome biogenesis disorder
ROS: Reactive oxygen species
SAE: Serious adverse event
SED: Single enzyme defect
SHP-1: Small heterodimer partner 1
THCA: Trihydroxycholestanoic acid
ULN: Upper limit of normal
ZS: Zellweger syndrome
ZSD: Zellweger spectrum disorder
